# Glandular differentiation in pT1 urothelial carcinoma of bladder predicts poor prognosis

**DOI:** 10.1038/s41598-019-41844-4

**Published:** 2019-03-29

**Authors:** Guobin Zhao, Chao Wang, Yuhong Tang, Xin Liu, Zihao Liu, Gang Li, Yanhui Mei

**Affiliations:** 1 Department of Urology, The First Affiliated Hospital of Hebei North University, Zhangjiakou City, Hebei Province 07500 China; 2 Department of Urology, Fifth Central Hospital of Tianjin, Tianjin, 300450 China; 3 College of Laboratory Medicine, Hebei North University, Zhangjiakou City, Hebei Province 07500 China; 4 Department of Urology, Tianjin Institute of Urology, The second hospital of Tianjin Medical University, Tianjin, 300211 China; 5 Department of Urology, Binzhou Medical University Hospital, Binzhou, 256603 China

## Abstract

To evaluate the effect of glandular differentiation (GD) on tumor recurrence and progression of pT1 bladder urothelial carcinoma (UC). We performed a retrospective analysis of 82 bladder urothelial carcinoma with glandular differentiation (UCGD) patients which was pathologically diagnosed as pT1, 166 patients of pT1 UC of bladder without histologic variants served as controls. Patients of UCGD were more likely to have higher recurrence (P = 0.002) rate and higher progression rate (P < 0.001). Moreover, UCGD and a poor 5 -year overall survival (OS) (P = 0.02) while there was no difference in cancer-specific survival (CSS) (P = 0.062) between two groups. According to univariate analysis, largest tumor size (HR 1.502, CI 1.158–1.861, P = 0.029), UCGD (HR 1.787, CI 1.298–2.552, P = 0.001), lymphovascular invasion (LVI) (HR 1.226, CI 1.013–1.945, P = 0.039). UCGD (HR 1.367, CI 1.115–1.853, P = 0.038) and LVI (HR 1.416, CI 1.120–2.254, P = 0.013) were prognostic factors associated with disease recurrence and progression, respectively. Additionally, Additionally, UCGD significantly influence disease recurrence (HR 1.871, CI 1.338–2.589, P < 0.001) and progression (HR 1.462, CI 1.138–2.393, p = 0.007). Similarly, LVI significantly influence disease recurrence (HR 1.356, CI 1.053–2.174, P = 0.042) and progression (HR 1.348, CI 1.052–1.944, p = 0.022) in multivariate analysis. UCGD is significantly associated with higher recurrence and progression rate in patients with newly diagnosed pT1. Recurrent cases should be performed radical cystectomy (RC) earlier.

## Introduction

Bladder cancer is one of the most common malignant tumor, being the sixth leading cause of new cancer cases and ninth leading cause of cancer-related mortality worldwide^1^. There is approximately 380,000 people newly being diagnosed of bladder cancer and 150,000 dead annually due to the tumor. Thus, any improvement to the tactics for diagnosing and treating bladder carcinoma seems to be important to the medical and public health^2,3^.

UC is the most common type in bladder cancer that accounts for nearly ninety percent. Meanwhile, UC has some morphological diversity which differ from conventional UC type^4,5^. GD which characterized by intratumoral tubular or enteric gland-like space, is one of the most common histological variant in UC^6^. Nowadays, an increasing number of studies had been focused on the impact of UC variants, but these conclusions remain contradictionary. Some reported that GD had more aggressive behavior than UC without differentiation, while others declared that the clinical outcomes make no difference^7–11^. Most conclusions referred above were not limited in pT1 UCGD, so the aim of our study is to assess the independent prognostic role of UCGD for newly diagnosed pT1 bladder UC case.

## Materials and Methods

### Patients selection

Between January 2004 to April 2016, we retrospectively analyzed the clinical and pathological information of pT1 UC patients in our hospitals and all cases were randomly selected. This study was approved by the ethics committee of First Affiliated Hospital of Hebei North University and the IRB NO was ZJK2017056. All human tumor tissue samples were carried out in accordance with relevant guidelines and ethical regulations. Moreover, informed consent was obtained from all subjects. Study inclusion criteria were (1) all patients performed transurethral resection of bladder tumor (TURBT) as initial treatment; (2) All tumors were pathologically diagnosed as pT1 stage and high grade UCGD or not. (3) All patients underwent intravesical chemotherapy regular with epirubicin/ hydroxycamptothecine and cystoscopy according to guidelines^12^. All cases received one immediate postoperative intravesical instillation and chemotherapy instillations regular once a week for eight weeks and then once a month last for one year. Bacillus Calmette-Guerin (BCG) was not used in our study because it has not been permitted by China Food and Drug Administrational. To avoid the influence of tumor grading, only high grade tumors were enrolled and all patients were divided into two cohorts by GD or not. Patients with other variant, a history of previous UC, concomitant upper tract UC or distant metastasis were excluded. Recurrence-free survival and progression-free survival were used to evaluate prognosis. The recurrence-free survival (RFS) period was counted from the time of first surgery to the date of first clinical recurrence. The progression-free survival (PFS) period was defined as from the day of surgery to the day of disease developed to higher histopathological staging.

### Pathology

**Figure 1:**
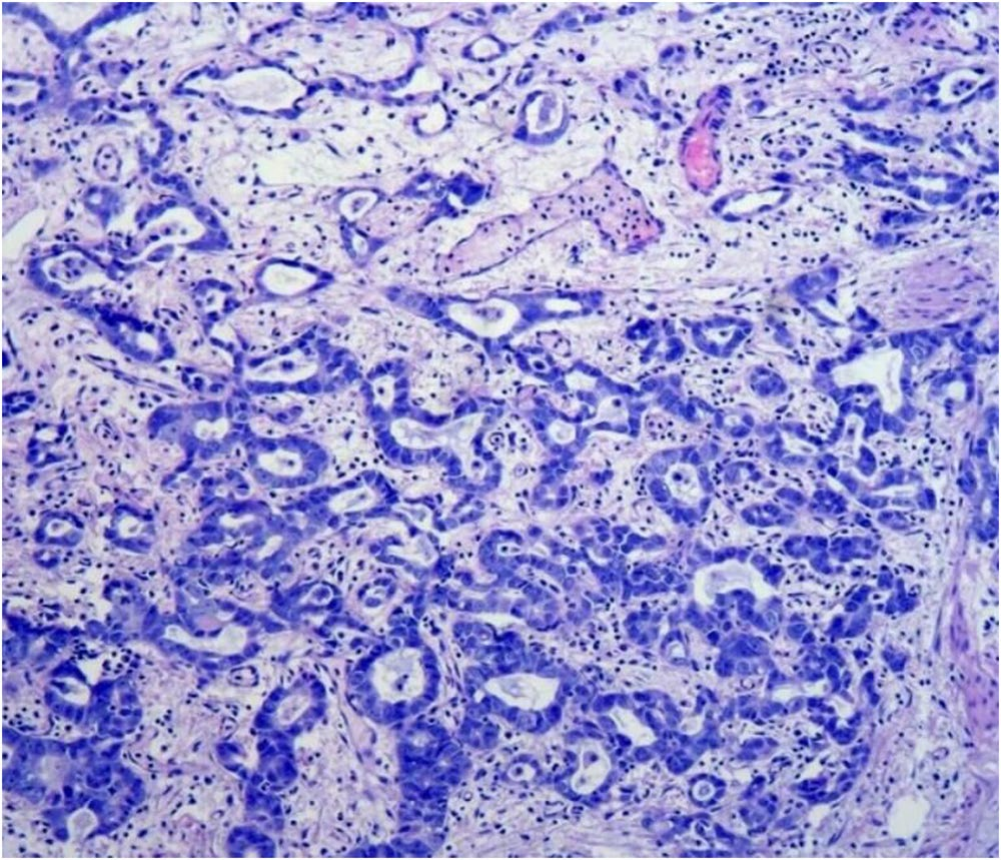
Urothelial carcinoma with glandular differentiation which tumor has intratumoral tubular or enteric gland-like space shape. Hematoxylin and Eosin staining Magnification, ×200.

Pathologic stage was determined according to the 2009 Union for International Cancer Control (UICC) TNM staging system, and grade was based on the 2004 World Health Organization (WHO) grading system for non-invasive urothelial neoplasia^13,14^. Diagnostic criteria over the research period kept invariant. Glandular differentiation was observed and confirmed by pathologists according to the morphology, including hematoxylin and eosin (H&E) staining (Fig. 1) and immunohistochemical staining (IHC).

### Statistical methods

The chi-square test were used to evaluate the association between pure urothelial carcinoma of bladder (PUCB) and UCGD. Kaplan-Meier method was used to calculate RFS, PFS, OS and CSS difference was determined by log-rank test. Univariate and multivariate Cox regression models were used to analyze predictive factors. All reported P values were two-sided and P < 0.05 was considered to have statistical significance. SPSS software (Version 22) was used to perform statistical analysis.

## Results

The clinicopathological data and demographics of patients with PUCB and those UCGD are shown in Table 1. The median age was 65.4(range 38 to 86) years old in patients with PUCB and 66.2(range 39 to 89) years old in patients of UCGD. There were no significance in terms of gender (P = 0.935), age (P = 0.707), tumor multiplicity (P = 0.419), tumor size (P = 0.850) and lymphovascular invasion (P = 0.081) in the two groups, but LVI were more common in UCGD (20.7%) than PUCB (12.6%).

**Table 1:** Clinical and pathological characteristics between pure UCB and UCGD.

Variable	UC(%)	UCGD(%)	P-value
Age(years)			0.707
<65	79(47.6)	37(45.1)	
≥65	87(52.4)	45(54.9)	
Gender			0.935
Male	137(82.5)	68(83.0)	
Female	29(17.5)	14(17.0)	
Largest tumor size (cm)			0.850
<3	119(71.8)	58(70.7)	
≥3	47(28.2)	24(29.3)	
Tumor multiplicity			0.419
Singal	110(66.3)	50(61.0)	
Multiple	56(33.7)	32(39.0)	
Lymphovascular invasion			0.081
Yes	21(12.6)	17(20.7)	
No	145(87.4)	65(79.3)	
Recurrence			0.005
Yes	77(46.6)	55(67.1)	
No	89(53.4)	27(32.9)	
Progression			0.007
Yes	24(14.6)	24(29.3)	
No	142(85.4)	58(70.7)	

Clinical and pathological characteristics of patients.

UC: urothelial carcinoma.

UCGD: urothelial carcinoma with glandular differentiation.

**Figure 2:**
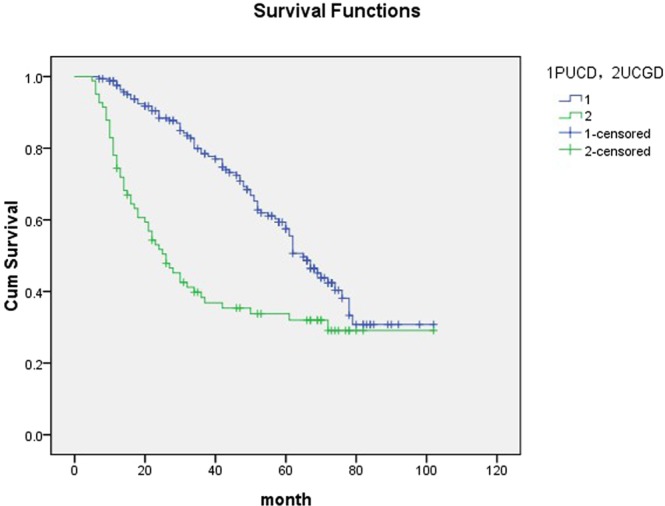
Kaplan-meier curve of the recurrence-free survival (RFS).

**Figure 3:**
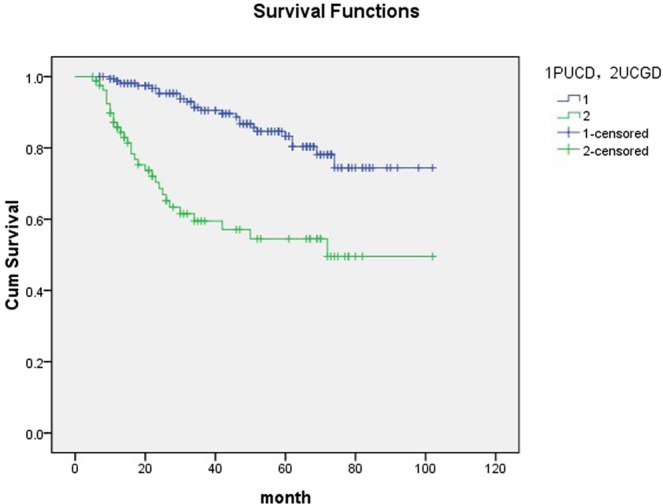
Kaplan-meier curve of the progression-free survival (PFS).

**Figure 4:**
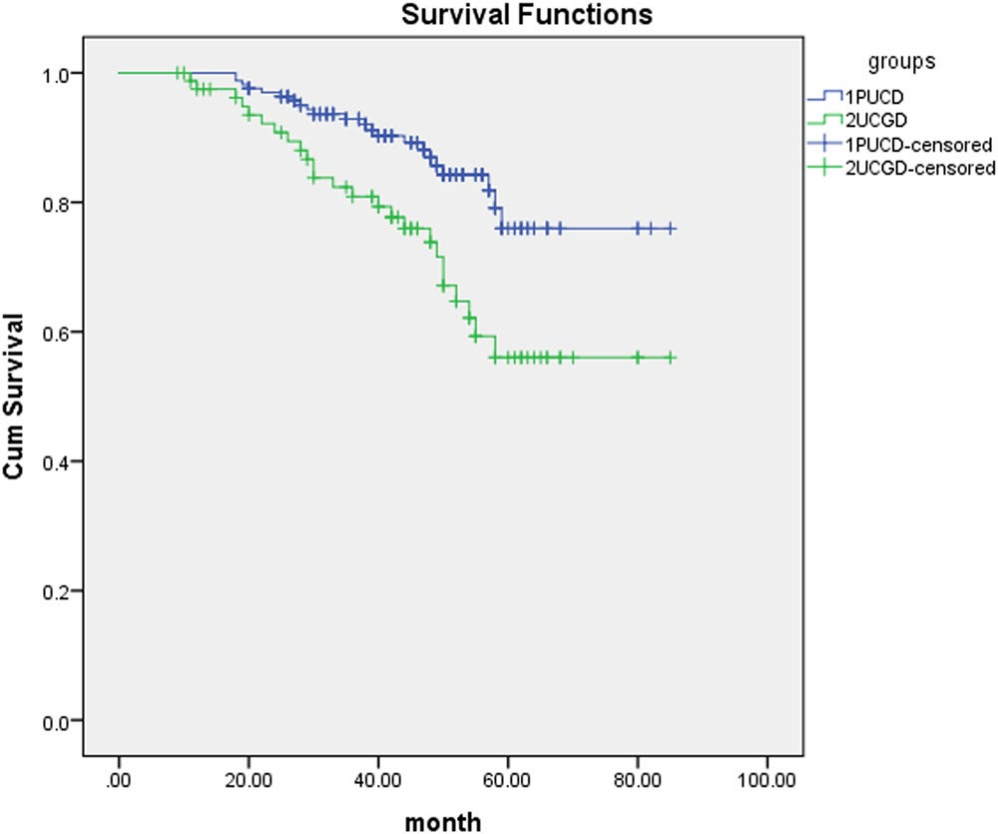
Kaplan-meier curve of the overall survival (OS).

**Figure 5:**
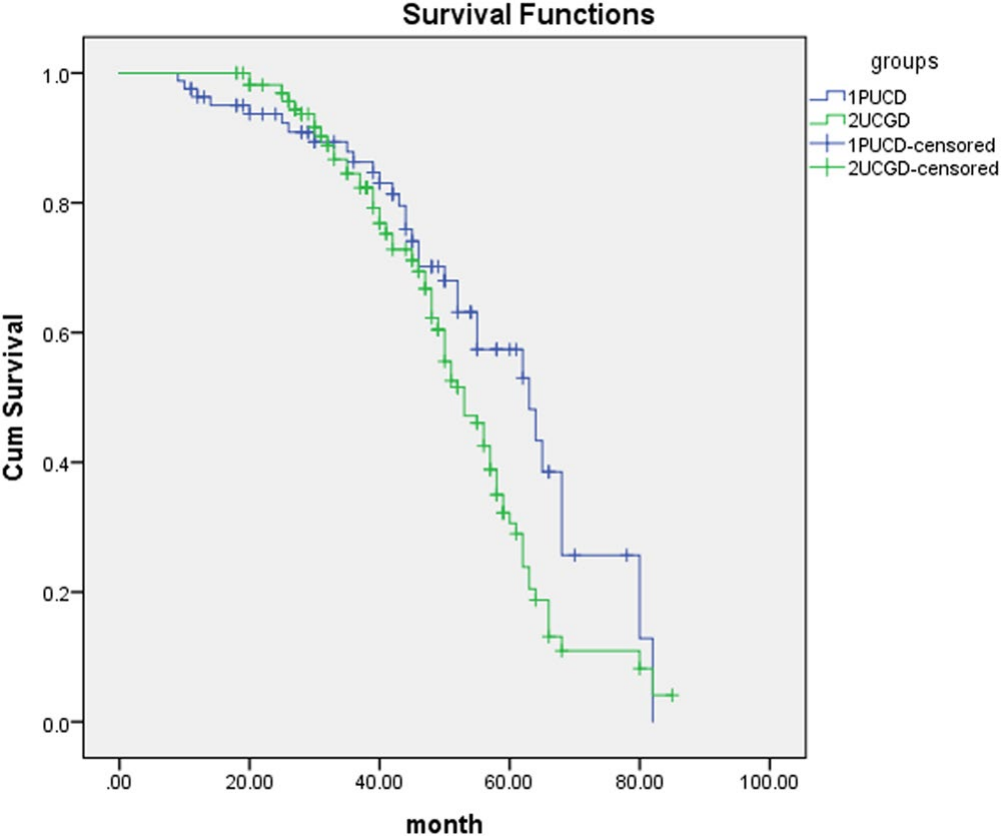
Kaplan-meier curve of the cancer-specific survival (CSS).

Mean follow-up time was 66.8 (6–107) months in the group of UCGD and 68.4 (7–102) months for the controls. Patients of UCGD were significantly more likely to recur than the PUCB group (67.1% versus 46.6%, P = 0.005). There was also a trend for patients with UCGD to have higher rate of progression comparing with patients with PUCB (29.3% versus 14.6%, P = 0.007). Further, we use Cox proportional hazard analysis for particular analysis (Tables 2 and 3). According to the results of univariate analysis, largest tumor size (HR1.502, 95% CI 1.158–1.861, P = 0.029), UCGD (HR 1.787, 95% CI 1.298–2.552, P = 0.001) and LVI (HR 1.226, 95% CI 1.013–1.945, P = 0.039) were associated with tumor recurrence. In multivariate analysis, LVI (HR 1.356, 95% CI 1.053–2.174, P = 0.042) and UCGD (HR 1.871, 95% CI 1.338–2.589, P < 0.001) were significantly influenced the recurrence. UCGD (HR 1.367, 95% CI 1.115–1.853, P = 0.038) and LVI (HR 1.416, 95% CI 1.120–2.254, P = 0.013) were associated with disease progression in univariate analysis, UCGD (HR 1.462, 95% CI 1.138–2.393, p = 0.007) and LVI (HR 1.348, 95% CI 1.052–1.944, p = 0.022) were the independent prognostic factors associated with progression in multivariate analysis. The Kaplan–Meier analysis was used to estimate RFS and PFS stratified by PUCB versus UCGD (Figs 2 and 3). Patients with UCGD had shorter mean RFS and PFS duration than those with PUCB. Moreover, UCGD and a poor 5 -year OS than PUCB (Fig. 4) while there was no difference in cancer-specific survival between two groups (Fig. 5).

**Table 2:** Univariate and multivariate Cox regression analysis of tumor recurrence.

Variable	Univariate analysis			Multivariate analysis		
	HR	95% CI	P	HR	95% CI	P
Largest tumor size	1.502	1.158–1.861	0.029	1.046	0.881–1.373	0.124
Tumor multiplicity	0.789	0.623–1.165	0.084	0.967	0.569–1.394	0.185
LVI	1.226	1.013–1.945	0.039	1.356	1.053–2.174	0.042
UCGD	1.787	1.298–2.552	0.001	1.871	1.338–2.589	<0.001

Univariate and multivariate Cox regression analyses according to tumor recurrence.

LVI: lymphovascular invasion.

UCGD: urothelial carcinoma with glandular differentiation.

HR: hazard ratio,

CI: confidence interval.

**Table 3:** Univariate and multivariate Cox regression analyses according to progression.

Variable	Univariate analysis			Multivariate analysis		
	HR	95% CI	P	HR	95% CI	P
Largest tumor size	1.360	0.961–1.994	0.059	1.236	0.897–1.676	0.083
Tumor multiplicity	0.883	0.524–1.226	0.154	0.952	0.674–1.234	0.069
LVI	1.416	1.120–2.254	0.013	1.348	1.052–1.944	0.022
UCGD	1.367	1.115–1.853	0.038	1.462	1.138–2.393	0.007

LVI: lymphovascular invasion.

UCGD: urothelial carcinoma with glandular differentiation.

HR: hazard ratio,

CI: confidence interval.

## Discussion

Urothelial carcinoma of bladder demonstrates a capacity of divergent histologic differentiation, which contains 13 kinds of variable types and glandular differentiation is the second most common type^15,16^. Kim^17^ reviewed the records of 1013 patients who underwent radical cystectomy, including 41 with glandular variant, the results revealed that the ten-year cancer specific survival didn’t significantly differ between the two groups(P = 0.71),but patients with glandular differentiation were more likely to have pT3–T4 tumors (P < 0.001) and pN+ disease (P = 0.05) than pure urothelial carcinoma. Patients of UCGD tended to have higher pathological grade and progression rate, which may indicate the aggressive clinical course^11^. We aimed to explore if UCGD had impact on tumor recurrence or progression, and so low grade tumor was not enrolled in order to eliminate the interference from tumor grade. Lee *et al*. reported glandular and squamous differentiation had effect of on oncologic outcomes, they found histologic variant to be an independent predictor for cancer specific survival on multivariate analysis^18^. Shanksalso reported that urothelial carcinoma of bladder with more than one variant tended to have higher rate of lymphatic metastasis^19^. However, to the best of our knowledge, the impact of GD on tumor recurrence and progression in pT1 patients of bladder cancer had not been reported. There was no paper focused on pure glandular differentiation of pT1 bladder tumor. Although Xu *et al*. found the correlation between squamous and/or glandular differentiation and RFS while not PFS which was different with our conclusions. There were several differences between our studies. First, both pTa and pT1 patients were all enrolled into Xu’s study while pure pT1 in our study. Second, squamous and/or glandular differentiation were all enrolled into Xu’s study while pure glandular differentiation in our study^20^. All these difference may contribute to different conclusions.

In our study, patients of UCGD were more likely to have higher recurrence (P = 0.005) rate and higher progression (P = 0.007) rate. Mille *et al*. conducted a research of 24 patients of UCGD, none of the UCGD type had transformed to bladder adenocarcinoma^21^. The diagnosis of bladder pure adenocarcinoma should eliminate any elements of urothelial carcinoma. Grignon *et al*. recommend that classify a tumor with mixed glandular and urothelial differentiation as urothelial carcinoma with glandular differentiation regardless of the extent of the glandular differentiation^22^. Recently, UCGD and bladder adenocarcinoma were considered two independent kinds of tumor and no relationships were found between them. Glandular tumor cells had variant shapes, which can be cubic or polygonous, forming tubular or enteric glands with mucin secretion^5,6^. Light microscopic evaluation is key to identify glandular lesions, but occasionally, it is difficult to distinguish GD from conventional UC or adenocarcinoma, particularly in cases with unclear features. In this case, usage of immunohistochemical (IHC) markers are helpful for definite diagnosis. It was reported that MUC5A, MUC2, CK20, c-erbB2 were completely positively expressed in tumor cells of UCGD, meanwhile, MUC1, CK7, 34βE12 could also be the markers helping distinguishing similar cells^23^.

A retrospectively analysis found that patients of upper urinary tract UC with squamous and/or glandular differentiation had worse outcomes^24^. Other researches have indicated that GD was associated with higher probability of recurrence^7,8,25^. In our study, UCGD was an independent prognostic factor associated with FRS in both univariate analysis (HR 1.787, 95% CI 1.298–2.552, p = 0.001) and multivariate analysis (HR 1.871, 95% CI 1.338–2.589, P < 0.001). LVI, largest tumor size were also related to disease recurrence in univariate analysis. All patients underwent intravesical chemotherapy with pharmorubicin, such results suggested that GD may be more resistant to chemotherapy.

Moreover, surgical specimens of patients of recurrence underwent re-TURBT or RC were further compared. UCGD were more likely to progress, univariate analysis and multivariate analyses showed an indicator of bladder cancer aggressiveness.

There are several limitations in our study. First, our data were retrospective and inherent biases occurred in patient selection and treatment. Second, owing to its relative scarcity, the number of pT1 UCGD patients was small. Further prospective studies including larger scale of patients are needed to value the significant role of glandular differentiation in pT1 patients.

## Conclusions

This study provides that pT1 UCGD is significantly associated with an increased risk of disease recurrence and progression. According to our study, recurrence of UCGD should be performed radical cystectomy (RC) routinely.
